# Therapeutic Effects of Conditioned Medium of Neural Differentiated Human Bone Marrow-Derived Stem Cells on Rotenone-Induced Alpha-Synuclein Aggregation and Apoptosis

**DOI:** 10.1155/2021/6658271

**Published:** 2021-01-22

**Authors:** Mahesh Ramalingam, Sujeong Jang, Han-Seong Jeong

**Affiliations:** Department of Physiology, Chonnam National University Medical School, Hwasun, Jeollanam-do 58128, Republic of Korea

## Abstract

Mesenchymal stem cells (MSCs) have been used against several diseases. Their potential mainly appears from its secreted biomolecules. Human bone marrow-derived stem cells (hBMSC) displayed neuronal functional characteristics after differentiation by basic fibroblast growth factor (bFGF) and forskolin. PD is a chronic age-related neurodegenerative disease (NDD) characterized by loss of dopaminergic neurons in the substantia nigra (SN) and abnormal accumulation of *α*-synuclein (*α*-syn) aggregations. In this present study, we evaluated the therapeutic effects of neural differentiated hBMSC (NI-hBMSC) conditioned medium (NI-hBMSC-CM) to a rotenone- (ROT-) induced Parkinson's disease (PD) model in SH-SY5Y cells. NI-hBMSC-CM treatment (50% diluted) in the last 24 h of 48 h ROT (0.5 *μ*M) toxicity showed a significant increase in cell survival. The decreased tyrosine hydroxylase (TH) expression as a hallmark of PD was increased by NI-hBMSC-CM. The Triton X-100-soluble and Triton X-100-insoluble cell lysate fractions were used in Western blotting. The oligomeric, dimeric, and monomeric phosphorylated serine129 (p-S129) *α*-syn and total monomeric *α*-syn were decreased during ROT toxicity in the Triton X-100-soluble fraction. The Triton X-100-insoluble fraction revealed that ROT toxicity significantly increased the oligomeric but decreased the dimeric and monomeric p-S129 *α*-syn expressions while all forms of total *α*-syn were increased in SH-SY5Y cells. NI-hBMSC-CM stabilized the physiological *α*-syn monomers and reduced aggregated insoluble p-S129 *α*-syn against ROT. The cytoskeletal proteins, neurofilament-H (NF-H), *β*3-tubulin (Tuj1), neuronal nuclei (NeuN), and synaptophysin (SYP) were significantly decreased during ROT toxicity. In addition, proapoptotic Bax was increased by ROT with decreased antiapoptotic Bcl-2 and Mcl-1 as well as proforms of caspase-9, caspase-3, caspase-7, and PARP-1. NI-hBMSC-CM ameliorated the neurotrophic protein expressions, controlled the Bax/Bcl-2 ratio, upregulated procaspases, and inactivated PARP-1. From our results, we conclude that NI-hBMSC-CM containing released biomolecules during neural differentiation employs regenerative effects on the ROT model of PD in SH-SY5Y cells.

## 1. Introduction

Mesenchymal stem cells (MSCs) can be found and isolated from many different body tissues, including bone marrow, placenta, adipose tissue [1], umbilical cord, bone trabeculae, muscle, synovium, dental pulp, and periodontal ligament [2]. The bone marrow-derived MSCs (BMSC) are the most studied due to their capacity to self-renew and proregenerative properties to differentiate into tissue-specific cells [3] such as osteoblasts, chondrocytes, adipocytes, hepatocytes, and neurons [4]. MSC are attractive therapeutic candidates in the treatment of several diseases including neurodegenerative diseases (NDD) [3, 5].

MSCs are able to cross the blood-brain barrier (BBB) [6]. However, *in vivo* MSC treatment has risks related to cell differentiation and their tumorigenic potential [7], and the consequent failure to reach the target site [8] or reach the injured site in the brain is negligible [9]. Evidence confirms that neuroprotection of MSC appears from its secretion of different proteins, including growth factors, cytokines, chemokines, metabolites, and bioactive lipids, which have paracrine and autocrine therapeutic activities [10, 11]. The secretome/conditioned medium (CM) from MSC (MSC-CM) is a heterogeneous bioactive molecule considered a biotechnological product, which is safer compared to the living MSC [5]. MSC-CM directly contributes to the recovery of the damaged tissues [11]. Therefore, considering their regenerative and restorative abilities, MSC-CM from different sources of MSC is proposed as the main biological effector as a possible alternative to MSC treatment in NDD [3, 12].

PD is a chronic NDD during aging mainly characterized by motor (bradykinesia, rigidity, and resting tremor) and nonmotor (depression, sleep disturbances, and memory deficits) complications due to the reduction of dopamine by degeneration of dopaminergic neurons in the substantia nigra pars compacta (SNpc) [13]. Additionally, PD is a highly complex and multifaceted disorder [14] including the presence of intraneuronal aggregates of the protein *α*-synuclein (*α*-syn), called Lewy bodies (LB) and Lewy neurites (LN) [15]. In neuronal cells, *α*-syn binds synaptic vesicles and plays a physiological role in stabilizing and modulating membrane structures and interactions at the presynapse [16]. *α*-syn is a 140 amino acid protein [17], and the monomeric *α*-syn has an amphiphatic N terminal, a hydrophobic nonamyloid component, and an acidic C terminal [18]. However, posttranslational modifications disrupt monomeric *α*-syn that leads to oligomerization and aggregation of *α*-syn [19]. Phosphorylation of the serine129 (p-S129) in the C terminus is highly thought to be a major marker of *α*-syn inclusions resembling the LBs in PD [20].

Rotenone (ROT), a piscicide from the roots and plants of genera *Lonchocarpus* and *Derris*, inhibits mitochondrial electron transport chain (ETC) complex 1 (EC 7.1.1.2; reduced nicotinamide adenine dinucleotide ubiquinone reductase; NADH ubiquinone oxidoreductase; type I NADH dehydrogenase) leading to reduced ATP production which induces oxidative stress by the formation of reactive oxygen species (ROS) [21]. It also reproduces the pathological hallmarks of PD such as a decreased nigrostriatal dopamine (DA) level by degeneration of tyrosine hydroxylase, *α*-syn phosphorylation and aggregation, and PD-like motor and nonmotor symptoms in culture cells and experimental animals [22]. These studies represent the administration of ROT as a neurotoxic agent to reproduce models of PD to analyze the effects of potential therapeutic agents in PD treatment.

Previously, our group reported that neural-induced hBMSC (NI-hBMSC) displayed the functional characteristics of neuronal cells in the presence of basic fibroblast growth factor (bFGF) and forskolin over two weeks [23, 24]. Therefore, further investigation with NI-hBMSC conditioned medium (NI-hBMSC-CM) may provide synergistic activities to achieve a multifactorial therapeutic effect in order to discover the molecular mechanism in the initiation and progression of PD. In this present study, we have evaluated the therapeutic effects of NI-hBMSC-CM on a ROT model of PD in human SH-SY5Y cells related to soluble/insoluble monomeric, dimeric, and oligomeric p-S129 and total *α*-syn as well as the specific cellular signaling pathways for understanding PD.

## 2. Materials and Methods

### 2.1. Preparation of hBMSC and Neurogenic Differentiation of NI-hBMSC

Human bone marrow was obtained from the mastoid process of healthy 29- to 51-year-old donors during mastoidectomy for ear surgery. Informed consent was obtained from ten donors according to the Guideline of the Ethics Committee of the Chonnam National University Medical School (Institutional Review Board No. I-2009-03-016). hBMSC were grown as adherent cultures in Dulbecco's modified Eagle's medium (DMEM; Hyclone, Logan, UT, USA) supplemented with 10% fetal bovine serum (FBS; Hyclone), 1% penicillin-streptomycin (Gibco BRL, Grand Island, NY, USA), and 0.2% amphotericin B (Gibco) in a 37°C humidified incubator with 5% CO_2_. For the experiment, hBMSC cells (passages 3-5) were maintained in DMEM containing 1% FBS for 7 days; then, the cell culture medium (hBMSC-CM) was aspirated, pooled, sterile filtered with 0.2 *μ*m syringe filter, and stored at -80°C until use.

To induce neurogenic differentiation, hBMSC (passages 3-5) were maintained in DMEM containing 1% FBS and supplemented with 100 ng/mL basic fibroblast growth factor (bFGF; Invitrogen Co., Carlsbad, CA, USA), for seven days, and then incubated in 10 *μ*M forskolin (Sigma Chemical Co., St. Louis, MO, USA) for the next seven days [23, 24]. Then, the neural-induced conditioned medium (NI-hBMSC-CM) was aspirated, pooled, sterile filtered with 0.2 *μ*m syringe filter, and stored at -80°C until use. We used multiple batches to collect the hBMSC-CM and NI-hBMSC-CM for our experiments.

### 2.2. SH-SY5Y Cell Culture and Rotenone Preparation

The human neuroblastoma cell line SH-SY5Y (RRID: CVCL_0019; ATCC® CRL-2266) was maintained in DMEM (Welgene Inc., Gyeonsangbuk-do, Republic of Korea) supplemented with 10% FBS and 1% penicillin-streptomycin at 37°C in a humidified atmosphere containing 5% CO_2_/95% air as previously described [25]. Confluent cultures (passages 15-22) were washed with phosphate-buffered saline (PBS), detached with 0.25% trypsin-EDTA solution, reseeded as 5 × 10^4^ cells/mL of DMEM containing 1% FBS, and used for experiments after overnight incubation.

ROT (Sigma R8875) stock was prepared at a concentration of 10 mM in solvent dimethyl sulfoxide (DMSO; Sigma D2650), aliquoted, stored at -80°C, and used within 6 months. Before starting each experiment, a ROT stock working solution was prepared by dilution with serum-free DMEM media.

### 2.3. Rotenone Toxicity and Treatments of hBMSC-CM or NI-hBMSC-CM

SH-SY5Y cells were incubated in the absence or presence of ROT (0.5 *μ*M) or solvent DMSO for 24 h. Then, media were removed, cells were treated with or without hBMSC-CM or NI-hBMSC-CM at 50% dilution in DMEM, and incubated in the absence or presence of ROT (0.5 *μ*M) for another 24 h (the experimental study plan was schemed in Supplementary Figure 2(a)). FBS was maintained at a concentration of 1% throughout the study. The phase contrast images were taken using an Olympus microscope (CKX41) equipped with a camera. Damaged and depleted floating cells in the medium and adherent cells detached by trypsinization were combined and subjected to the trypan blue cell counting method. The number of surviving cells were counted using a LUNA-II™ (Logos Biosystems, Gyeonggi-do, Republic of Korea) automated cell counter. The cell count assay was performed in triplicate and expressed as a percentage (%) of the control.

### 2.4. Preparation of Total Cell Lysates and Immunoblotting

After 48 h, cells were harvested by scraping with media by a cell scraper, pelleted, and washed twice with PBS, then exposed to cell lysis buffer (100 mM Tris-HCl (pH 7.6), 100 mM NaCl, 1% Nonidet P-40, 1% sodium deoxycholate, 0.1% sodium dodecyl sulfate (SDS), and 1% Triton X-100) supplemented with protease and phosphatase inhibitors and incubated for 30 min in ice. Lysates were centrifuged at 13,200 rpm for 15 min at 4°C, and the supernatants were collected as to total cell lysate. Protein concentrations were determined by using the BCA Protein Assay Kit (Thermo Fisher Scientific, Waltham, MA, USA #23225) following the manufacturer's instructions. Proteins (15 *μ*g) were separated on 8–14% SDS-polyacrylamide gels and transferred onto nitrocellulose membranes (Millipore, Berlington, MA, USA, HATF00010). The membranes were washed with PBS containing 0.5% (*v*/*v*) Tween 20 (PBS-T) followed by blocking with 5% (*v*/*v*) nonfat dried milk solution prepared in PBS-T and then incubated overnight with primary antibodies at 4°C. The antibodies used are listed in Supplementary Table 1. After this, the membranes were exposed to secondary antibodies conjugated to horseradish peroxidase for 2~3 h at room temperature (RT) and washed thrice with PBS-T. The signals were detected by an enhanced chemiluminescence (ECL) system (Millipore, WBLUR0500) using a LAS 4000 luminescent image analyzer (GE Healthcare, Japan). The membranes were kept in Western blot stripping buffer (Thermo Fisher Scientific, #21059) with constant shaking for 60 min. Equal protein loading was assessed by the expression level of *β*-actin or GAPDH. Densitometric analysis was performed using ImageJ (National Institutes of Health, Bethesda, MD, United States) software.

### 2.5. Triton X-100-Soluble and Triton X-100-Insoluble Fractionation and Western Blotting of *α*-syn

After 48 h of experiments, SH-SY5Y cells were lysed on ice in cell lysis buffer containing protease and phosphatase inhibitors with 1% Triton X-100 as mentioned above for 30 min. Lysates were centrifuged at 13,200 rpm for 15 min at 4°C, and the supernatants were collected as Triton X-100-soluble fraction. The cell pellets were washed with PBS then dissolved in the cell lysis buffer containing protease and phosphatase inhibitors with 1% Triton X-100 and 2% SDS and sonicated for 10 s and used as Triton X-100-insoluble fraction. Protein concentrations were determined by using the BCA Protein Assay Kit. Equal amounts of proteins (30 *μ*g) were separated on 8 or 12% SDS-polyacrylamide gels and transferred onto nitrocellulose membranes. Immediately after the transfer, the membranes were prefixed with 4% paraformaldehyde (PFA; GeneAll Biotechnology, Seoul, Republic of Korea, SM-P-01-100) in PBS containing 0.01% glutaraldehyde (Sigma 340855) for 60 min at RT and then washed with PBS. Blocking was performed with 5% skim milk in Tris-buffered saline (TBS) with 0.1% Tween-20 (TBS-T) for 60 min. Membranes were then incubated with anti-p-S129 *α*-syn (Abcam, Cambridge, United Kingdom, ab51253) primary antibody in blocking buffer overnight at 4°C. The membranes were then washed 3 × 10 min in TBS and incubated with the secondary antibody in blocking buffer followed by washing 3 × 10 min in TBS and developing with ECL. After p-S129 *α*-syn visualization, the membranes were washed with PBS-T and kept in Western blot stripping buffer with constant shaking for 60 min, washed 3 × 10 min in PBS-T, prefixed with 4% PFA in PBS for 60 min at RT, and then rinsed with PBS. Blocking was in 5% skim milk in TBS-T for 60 min. The membranes were then incubated in total *α*-syn (Abcam ab212184) primary antibody in blocking buffer overnight at 4°C. Subsequently, the membranes were then washed 3 × 10 min in TBS and incubated in secondary antibody in blocking buffer followed by washing 3 × 10 min in TBS and developing with ECL. Equal protein loading was assessed by the expression level of GAPDH, which was used as an internal control. Densitometric analysis was performed using ImageJ software.

### 2.6. Statistical Analysis

Data are expressed as the mean ± standard error mean (SEM) from three independent experiments in SH-SY5Y cells treated with different batches of hBMSC-CM or NI-hBMSC-CM. The significance level of treatment effects was determined using one-way analysis of variance (ANOVA) followed by Tukey's *post hoc* multiple comparison test. A probability of <5% (*p* < 0.05) was considered to be statistically significant. GraphPad Prism® 5.0 software (GraphPad Software Inc.) was used for data analyses and preparation of all graphs.

## 3. Results

### 3.1. NI-hBMSC-CM on Rotenone-Induced Death in SH-SY5Y Cells

**Figure 1:**
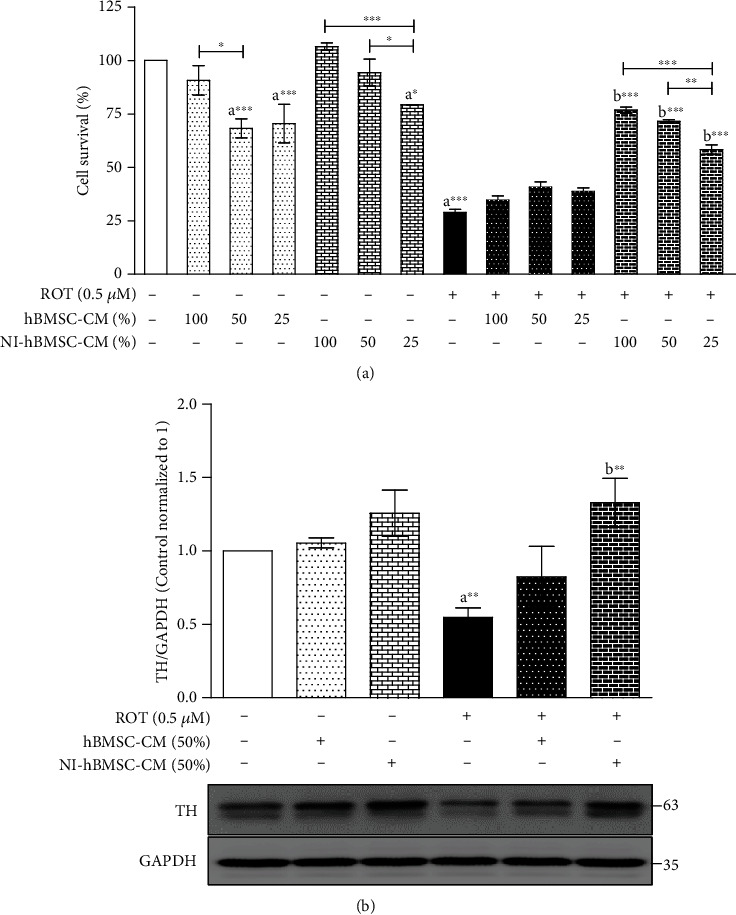
(a) SH-SY5Y cells incubated with the absence or presence of ROT (0.5 *μ*M) for 48 h were treated with hBMSC-CM or NI-hBMSC-CM at 100 or 50 or 25% during the last 24 h, and cell survival was assessed by the trypan blue assay. Data are the mean ± SEM of three independent experiments and analyzed by one-way analysis of variance (ANOVA) followed by Tukey's *post hoc* test. Statistical significance: ^a^compared with control; ^b^compared with ROT; ^∗^*p* < 0.05 and ^∗∗∗^*p* < 0.001. (b) Cells incubated with the absence or presence of ROT (0.5 *μ*M) for 48 h were treated with hBMSC-CM (50%) or NI-hBMSC-CM (50%) during the last 24 h and assessed for TH and GAPDH by Western blotting. Each picture is representative of three independent experiments. Data are the mean ± SEM of three independent experiments and analyzed by one-way analysis of variance (ANOVA) followed by Tukey's *post hoc* test. Statistical significance: ^a^compared with control; ^b^compared with ROT; ^∗∗^*p* < 0.01.

The cell survival rate was gradually decreased with increasing concentrations of ROT revealing that ROT dose- and time-dependently increased cell death after 24 and 48 h (data not shown). Based on that, ROT at the concentration of 0.5 *μ*M for 48 h was used in all subsequent experiments. SH-SY5Y cells were exposed with or without ROT (0.5 *μ*M) for 24 h; then, the culture medium was removed; a new culture medium with or without hBMSC-CM or NI-hBMSC-CM at 100, 50, and 25% was diluted in DMEM +1% FBS incubated in the absence or presence of ROT (0.5 *μ*M) for another 24 h (Figure 1(a)). NI-hBMSC-CM treatment with 100, 50, and 25% dilution against ROT toxicity showed significantly increased cell numbers; on the contrary, treatment of hBMSC-CM did not show any significant changes against ROT toxicity. For the treatment to control cells without ROT toxicity, NI-hBMSC-CM showed no significant changes at 100 and 50% but decreased at 25% dilution. hBMSC-CM at 50 and 25% dilution also showed a significant decrease in cell numbers against the control group (Figure 1(a)). Morphological images showed that ROT toxicity reduced the cell numbers and changed the cell surface compared with the control group. Treatments with NI-hBMSC-CM increased cell numbers (Supplementary Figure 1). From these results, NI-hBMSC-CM at 50% dilution was selected for further study to evaluate its therapeutic effects. The experimental study plan was prepared (Supplementary Figure 2(a)), and the morphological changes were observed (Supplementary Figure 2(b)).

### 3.2. NI-hBMSC-CM on ROT-Induced TH Protein Expression in SH-SY5Y Cells

Tyrosine hydroxylase (TH), the rate-limiting enzyme for the biosynthesis of dopamine and a specific marker for PD, was evaluated by the Western blotting method (Figure 1(b); Supplementary Figure 5). ROT toxicity for 48 h significantly decreased (*p* < 0.01) the TH protein expression suggesting that ROT induced the dopaminergic neurodegeneration as a hallmark of PD. As expected, the NI-hBMSC-CM treatment at the last 24 h showed increased TH expression (*p* < 0.01) against 48 h of ROT toxicity. hBMSC-CM showed a nonsignificant increase in TH expression (*p* > 0.05). These results revealed the therapeutic efficiency of NI-hBMSC-CM on neuroprotection against ROT-induced PD in SH-SY5Y cells.

### 3.3. NI-hBMSC-CM on ROT-Induced p-S129 and Total *α*-syn Protein Expressions in SH-SY5Y Cells

**Figure 2:**
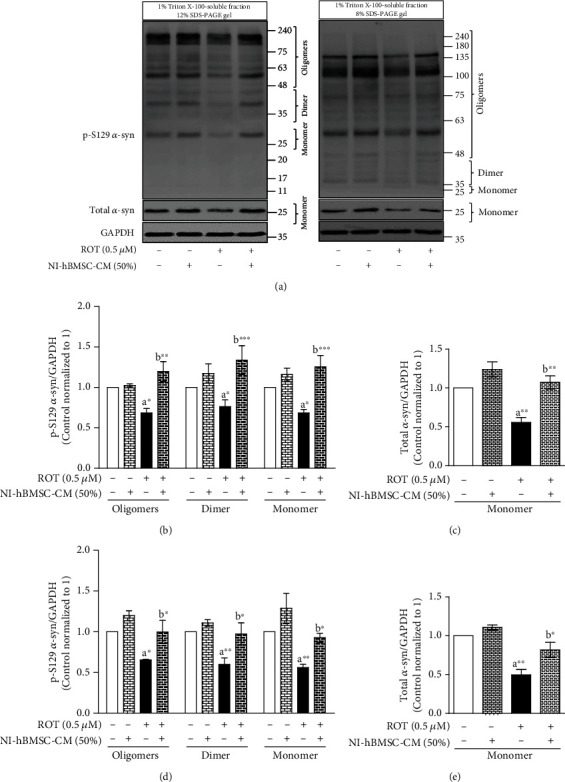
SH-SY5Y cells were seeded as 5 × 10^4^ cells/mL of DMEM containing 1% FBS and used for experiments after overnight incubation. Cells incubated with the absence or presence of ROT (0.5 *μ*M) for 48 h were treated with NI-hBMSC-CM (50%) during the last 24 h. Cell lysates were prepared as 1% Triton X-100-soluble and Triton X-100-insoluble (2x SDS soluble) fractions. p-S129 and total *α*-syn were analyzed from 1% Triton X-100-soluble fractions by Western blotting using 12% and 8% SDS-PAGE gels (a). Each picture is representative of three independent experiments. Bar graphs represent fold changes in p-S129 *α*-syn/GAPDH (b, d) and total *α*-syn/GAPDH (c, e) in SDS-PAGE gels of 12% (b, c) or 8% (d, e). Data are the mean ± SEM of three independent experiments and analyzed by one-way analysis of variance (ANOVA) followed by Tukey's *post hoc* test. Statistical significance: ^a^compared with control; ^b^compared with ROT; ^∗^*p* < 0.01, ^∗∗^*p* < 0.05, and ^∗∗∗^*p* < 0.001.

**Figure 3:**
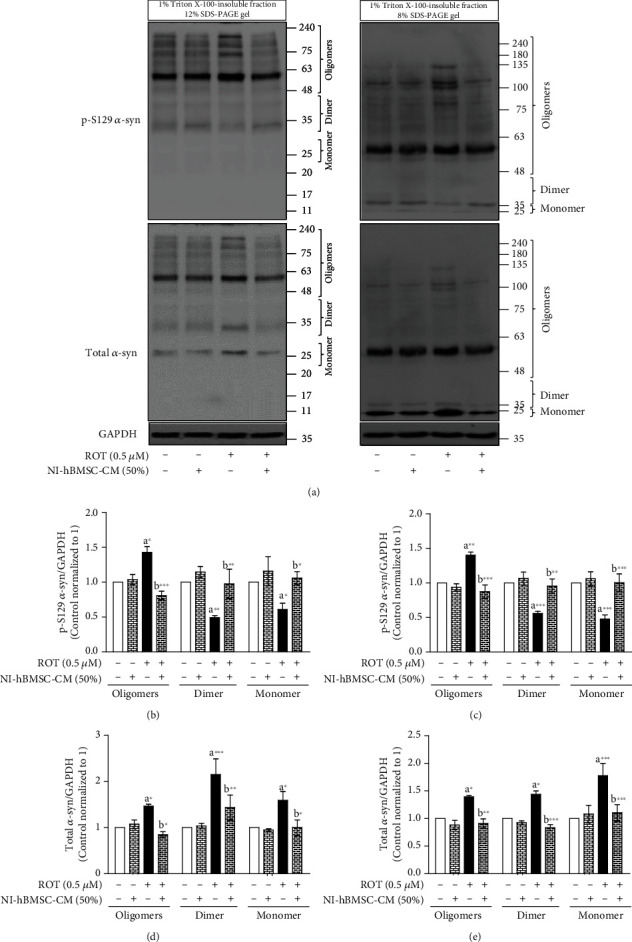
SH-SY5Y cells were seeded as 5 × 10^4^ cells/mL of DMEM containing 1% FBS and used for experiments after overnight incubation. Cells incubated with the absence or presence of ROT (0.5 *μ*M) for 48 h were treated with NI-hBMSC-CM (50%) during the last 24 h. Cell lysates were prepared as 1% Triton X-100-soluble and Triton X-100-insoluble (2x SDS soluble) fractions. p-S129 and total *α*-syn were analyzed from 1% Triton X-100-insoluble (2x SDS soluble) fractions by Western blotting using 12% and 8% SDS-PAGE gels (a). Each picture is representative of three independent experiments. Bar graphs represent fold changes in p-S129 *α*-syn/GAPDH (b, d) and total *α*-syn/GAPDH (c, e) in SDS-PAGE gels of 12% (b, c) or 8% (d, e). Data are the mean ± SEM of three independent experiments and analyzed by one-way analysis of variance (ANOVA) followed by Tukey's *post hoc* test. Statistical significance: ^a^compared with control; ^b^compared with ROT; ^∗^*p* < 0.05, ^∗∗^*p* < 0.01, and ^∗∗∗^*p* < 0.001.

To assess the ROT-induced *α*-syn-mediated toxicity in SH-SY5Y cells, Western blot analyses of the Triton X-100-soluble and Triton X-100-insoluble cell lysate fractions were used to detect the protein expressions of p-S129 and total *α*-syn (Figures 2 and 3). The protein lysate fractions were loaded onto 12% and 8% SDS-PAGE gels and immunoblotted to detect oligomeric, dimeric, and monomeric forms of p-S129 and total *α*-syn accumulation.

From Figure 2(a) and Supplementary Figure 6, ROT (0.5 *μ*M for 48 h) toxicity significantly decreased the oligomeric (*p* < 0.05 in 12 and 8% SDS-PAGE gels), dimeric, and monomeric (both with *p* < 0.05 in 12% SDS-PAGE gel; *p* < 0.01 in 8% SDS-PAGE gel) forms of p-S129 *α*-syn (Figures 2(b) and 2(d)) as well as total monomeric *α*-syn (*p* < 0.01 in 12 and 8% SDS-PAGE gels (Figures 2(c) and 2(e)) in the Triton X-100-soluble cell lysate fraction. In NI-hBMSC-CM treatment, p-S129 and total *α*-syn were significantly increased compared with ROT toxicity only. The oligomeric (*p* < 0.01 in 12% SDS-PAGE gel; *p* < 0.05 in 8% SDS-PAGE gel), dimeric, and monomeric (both with *p* < 0.001 in 12% SDS-PAGE gel; *p* < 0.05 in 8% SDS-PAGE gel) forms of p-S129 *α*-syn as well as total monomeric *α*-syn (*p* < 0.01 in 12% SDS-PAGE gel; *p* < 0.05 in 8% SDS-PAGE gel) were increased. Additionally, the ratio of monomeric p-S129/total *α*-syn was not changed during ROT-induced toxicity and NI-hBMSC-CM treatment (Supplementary Figures 3(a) and 3(b)) suggesting that p-S129 expressions were positively correlated with their total *α*-syn levels.

Triton X-100-insoluble p-S129 protein expressions with its total *α*-syn expressions are shown in Figure 3(a) and Supplementary Figure 7. ROT toxicity induced an increase in oligomeric (*p* < 0.05 in 12% SDS-PAGE gel, Figure 3(b); *p* < 0.01 in 8% SDS-PAGE gel, Figure 3(c)) but decreased in dimeric (*p* < 0.01 in 12% SDS-PAGE gel; *p* < 0.001 in 8% SDS-PAGE gel) and monomeric (*p* < 0.05 in 12% SDS-PAGE gel; *p* < 0.001 in 8% SDS-PAGE gel) p-S129 *α*-syn expressions. NI-hBMSC-CM treatment significantly returned the oligomeric (*p* < 0.001 in 12 and 8% SDS-PAGE gels), dimeric (*p* < 0.01 in 12% and 8% SDS-PAGE gel), and monomeric (*p* < 0.05 in 12% SDS-PAGE gel; *p* < 0.001 in 8% SDS-PAGE gels) p-S129 protein expressions induced by ROT. Moreover, total *α*-syn of Triton X-100-insoluble fractions were significantly increased in oligomeric, dimeric, and monomeric forms (*p* < 0.05, *p* < 0.001, and *p* < 0.05 in 12% SDS-PAGE gel, Figure 3(d); *p* < 0.05, *p* < 0.05, and *p* < 0.001 in 8% SDS-PAGE gel, Figure 3(e)) in ROT-induced toxicity as compared with the control levels. NI-hBMSC-CM treatment to ROT toxicity for the last 24 h of 48 h reduced the oligomeric (*p* < 0.05 in 12% SDS-PAGE gel; *p* < 0.01 in 8% SDS-PAGE gel), dimeric (*p* < 0.01 in 12% SDS-PAGE gel; *p* < 0.001 in 8% SDS-PAGE gel), and monomeric (*p* < 0.05 in 12% SDS-PAGE gel; *p* < 0.001 in 8% SDS-PAGE gel) forms of total *α*-syn (Figures 3(d) and 3(e)). In addition, the ratio of oligomeric p-S129/total *α*-syn in ROT-induced toxicity and NI-hBMSC-CM treatment to ROT was not changed (Supplementary Figures 3(c) and 3(d)) suggesting that insoluble oligomers of p-S129 expressions were positively correlated with those of total *α*-syn oligomeric levels. The ratio of dimeric and monomeric p-S129/total *α*-syn in ROT-induced toxicity was decreased significantly (*p* < 0.001 in all), and NI-hBMSC-CM treatment significantly increased to control levels during ROT toxicity (Supplementary Figures 3(c) and 3(d)).

### 3.4. NI-hBMSC-CM on ROT-Induced Protein Expressions of Neuronal Markers in SH-SY5Y Cells

**Figure 4:**
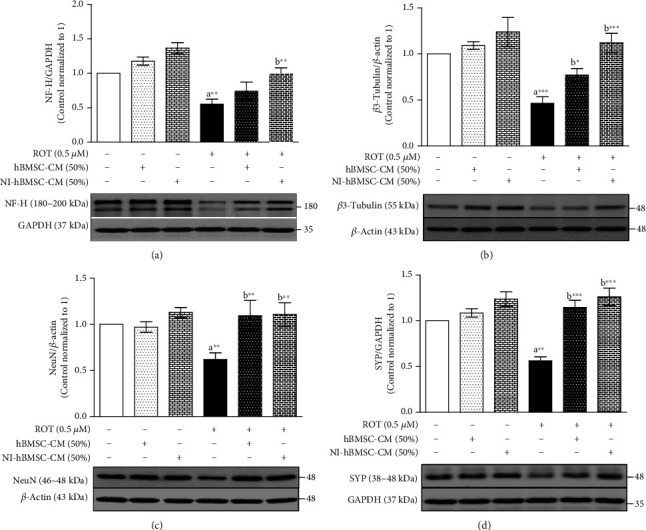
SH-SY5Y cells were seeded as 5 × 10^4^ cells/mL of DMEM containing 1% FBS and used for experiments after overnight incubation. Cells with the absence or presence of ROT (0.5 *μ*M) for 48 h were treated with hBMSC-CM (50%) or NI-hBMSC-CM (50%) during the last 24 h and analyzed for NF-H (a), *β*3-tubulin (b), NeuN (c), SYP (d), and GAPDH or *β*-actin by Western blotting. Each picture is representative of three independent experiments. Data are the mean ± SEM of three independent experiments and analyzed by one-way analysis of variance (ANOVA) followed by Tukey's *post hoc* test. Statistical significance: ^a^compared with control; ^b^compared with ROT; ^∗^*p* < 0.05, ^∗∗^*p* < 0.01, and ^∗∗∗^*p* < 0.001.

The phosphorylation and oligomerization of *α*-syn can interact with several neuronal markers such as neurofilament-heavy (NF-H), *β*3-tubulin (Tuj1), neuronal nuclei (NeuN), and synaptophysin (SYP). As expected, NF-H (*p* < 0.01; Figure 4(a)), *β*3-tubulin (*p* < 0.001; Figure 4(b)), NeuN (*p* < 0.01; Figure 4(c)), and SYP (*p* < 0.01; Figure 4(d)) protein expressions were significantly decreased in the ROT toxicity group compared with the control group. Meanwhile, NI-hBMSC-CM treatment significantly increased these protein expressions (NF-H and NeuN: *p* < 0.01; *β*3-tubulin and SYP: *p* < 0.001) in SH-SY5Y cells during ROT toxicity. hBMSC-CM treatment also increased the *β*3-tubulin (*p* < 0.05), NeuN (*p* < 0.01), and SYP (*p* < 0.001) expressions against ROT toxicity. Treatment of NI-hBMSC-CM or hBMSC-CM to control cells did not show any changes (Figure 4 and Supplementary Figure 8).

### 3.5. NI-hBMSC-CM on ROT-Induced Apoptotic Protein Expressions in SH-SY5Y Cells

**Figure 5:**
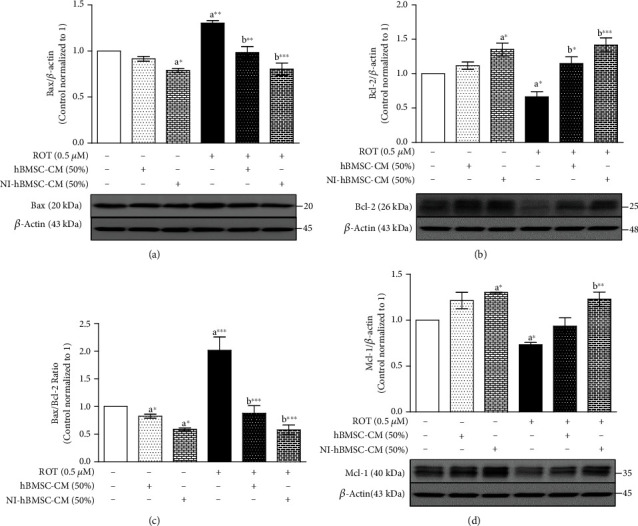
SH-SY5Y cells were seeded as 5 × 10^4^ cells/mL of DMEM containing 1% FBS and used for experiments after overnight incubation. Cells with the absence or presence of ROT (0.5 *μ*M) for 48 h were treated with hBMSC-CM (50%) or NI-hBMSC-CM (50%) during the last 24 h and assessed for Bax (a), Bcl-2 (b), Bax/Bcl-2 ratio (c), Mcl-1 (d), and *β*-actin by Western blotting. Each picture is representative of three independent experiments. Data are the mean ± SEM of three independent experiments and analyzed by one-way analysis of variance (ANOVA) followed by Tukey's *post hoc* test. Statistical significance: ^a^compared with control; ^b^compared with ROT; ^∗^*p* < 0.05, ^∗∗^*p* < 0.01, and ^∗∗∗^*p* < 0.001.

We next detected the levels of proapoptotic Bax, antiapoptotic Bcl-2, and Mcl-1 protein expressions to examine the effects of NI-hBMSC-CM on the expression of apoptosis-related proteins in ROT-induced SH-SY5Y cells (Figure 5 and Supplementary Figure 9). ROT toxicity for 48 h significantly increased Bax (*p* < 0.01; Figure 5(a)), while decreasing Bcl-2 (*p* < 0.05; Figure 5(b)) and Mcl-1 (*p* < 0.05; Figure 5(d)) protein expression levels compared to the control group. However, treatment with NI-hBMSC-CM or hBMSC-CM significantly inhibited the Bax levels (*p* < 0.001 by NI-hBMSC-CM; *p* < 0.01 by hBMSC-CM) with increasing Bcl-2 (*p* < 0.001 by NI-hBMSC-CM; *p* < 0.05 by hBMSC-CM) and Mcl-1 (*p* < 0.01 by NI-hBMSC-CM) levels against ROT-induced SH-SY5Y cells. Notably, NI-hBMSC-CM treatment to control cells showed decreased Bax (*p* < 0.05) and increased Bcl-2 and Mcl-1 (both *p* < 0.05) protein expressions compared to untreated control SH-SY5Y cells. Bcl-2 as an antiapoptotic member of the Bcl-2 family can bind to Bax to form Bcl-2:Bax heterodimers, thereby attenuating the apoptotic effect of Bax. Apparently, ROT caused an increase in the Bax/Bcl-2 ratio (*p* < 0.001; Figure 5(c)) while decreasing the Bcl-2/Bax ratio (*p* < 0.001; Supplementary Figure 4(a)). Treatment with NI-hBMSC-CM or hBMSC-CM significantly decreased the Bax/Bcl-2 ratio (*p* < 0.001; Figure 5(c)) with increasing the Bcl-2/Bax ratio (*p* < 0.001; Supplementary Figure 4(a)) against ROT-induced and untreated control SH-SY5Y cells.

**Figure 6:**
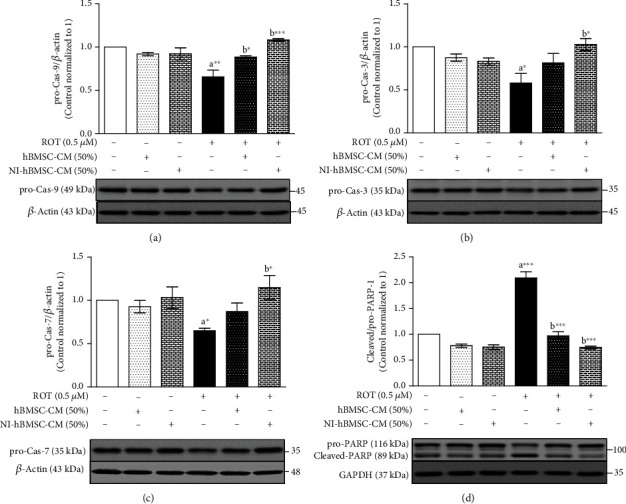
SH-SY5Y cells were seeded as 5 × 10^4^ cells/mL of DMEM containing 1% FBS and used for experiments after overnight incubation. Cells with the absence or presence of ROT (0.5 *μ*M) for 48 h were treated with hBMSC-CM (50%) or NI-hBMSC-CM (50%) during the last 24 h and assessed for pro-Cas-9 (a), pro-Cas-3 (b), pro-Cas-7 (c), PARP (d), and *β*-actin or GAPDH by Western blotting. Each picture is representative of three independent experiments. Data are the mean ± SEM of three independent experiments and analyzed by one-way analysis of variance (ANOVA) followed by Tukey's *post hoc* test. Statistical significance: ^a^compared with control; ^b^compared with ROT; ^∗^*p* < 0.05, ^∗∗^*p* < 0.01, and ^∗∗∗^*p* < 0.001.

In Figure 6 and Supplementary Figure 10, the pro-Cas-9, pro-Cas-3, and pro-Cas-7 expressions were markedly downregulated (*p* < 0.01, Figure 6(a); *p* < 0.05, Figure 6(b); and *p* < 0.05, Figure 6(c), respectively) in the ROT toxicity group. However, treatment with NI-hBMSC-CM significantly increased the expressions of pro-caspases (Cas-9: *p* < 0.001; Cas-3 and Cas-7: *p* < 0.01) against ROT-induced apoptosis. hBMSC-CM treatment also increased the pro-Cas-9 (*p* < 0.05) level against ROT toxicity. Moreover, the pro-PARP-1 level was decreased (*p* < 0.05; Supplementary Figure 4(b)), but the level of cleaved-PARP-1 (*p* < 0.001; Supplementary Figure 4(c)) along with the cleaved/pro-PARP-1 ratio (*p* < 0.001; Figure 6(d)) was increased in the ROT-induced PD model in SH-SY5Y cells. NI-hBMSC-CM treatment increased the pro-PARP-1 (*p* < 0.05) level along with a decreased cleaved-PARP-1 (*p* < 0.001) and cleaved/pro-PARP-1 ratio (*p* < 0.001; Figure 6(d)) in ROT toxicity. hBMSC-CM treatment decreased the cleaved-PARP-1 level (*p* < 0.001) with a decreased cleaved/pro-PARP-1 ratio (*p* < 0.001) in ROT-induced toxicity in SH-SY5Y cells. These results demonstrated that NI-hBMSC-CM inhibited the ROT-induced apoptosis in SH-SY5Y cells.

## 4. Discussion

MSCs are widely distributed and easily obtained from the body tissues assured by their self-renewal abilities and regeneration-mediated therapeutic approaches in managing numerous diseases [26, 27]. We previously reported the isolation and culture of hBMSC that expressed more than 95% MSC-specific markers such as CD13, CD44 (endoglin), CD90 (Thy-1), or CD166 but did not express markers for hematopoietic stem cells, including CD14, CD34, and CD45. The astrocyte marker (GFAP) and neuronal markers (NF-L, NF-M, NF-H, MAP2, NeuN, and Tuj1) were very low or undetectable in hBMSC [23].

After neuronal differentiation with bFGF and forskolin supplements for two weeks, the majority of NI-hBMSC exhibited bipolar or multipolar morphologies with branched processes, and the neuronal marker-positive neurons were considerably increased in larger numbers as compared with GFAP-positive astrocytes. Using the patch-clamp technique in whole-cell configuration, more than 66% of the NI-hBMSC expressed voltage-dependent sodium currents compared with hBMSC exhibiting virtually no sodium current. The mRNA expression levels of MaxiK (responsible for human large-conductance-, voltage-, and calcium-dependent K^+^ channel); Kv1.4, Kv4.2, and Kv4.3 (responsible for human voltage-dependent K^+^ channel); Eag1 and Eag2 (responsible for human ether-à-go-go K^+^ channel); NE-Na (responsible for TTX-sensitive Na^+^ channel); CACNA1C (responsible for human voltage-dependent L-type Ca^2+^ channel, alpha 1C subunit); and CACNA1G (responsible for human voltage-dependent T-type Ca^2+^ channel, alpha 1G subunit) in NI-hBMSC were significantly increased [23]. mRNA gene expressions of Wnt4, Wnt5a, Wnt11, and Fzd3 were increased with increased protein expressions of Wnt4 and Wnt5a by upregulated JNK-related proteins in NI-hBMSC suggesting that JNK is an important modulator in neurogenic differentiation [24]. In addition, transplantation of NI-hBMSC into the neomycin-treated deafened guinea pig cochlea exhibited a significant increase in the number of spiral ganglion neurons (SGNs) concluding the potential of NI-hBMSC in hearing loss mammals [23].

The cell-to-cell contacts as well as the immune-modulatory secreted factors attributed to the mechanism of actions to regenerate injured cells and tissues [26]. Secretome or conditioned medium (CM) contains a set of bioactive factors/molecules including lipids, proteins, nucleic acids, chemokines, cytokines, growth factors, hormones, and extracellular vesicles released from cell, tissue, or organism [28–30]. MSC secreted bioactive molecules released into their surrounding medium having therapeutic roles on cellular migration, proliferation, immunomodulation, and tissue regeneration [26, 31]. The neural differentiation to human adipose tissue- or bone marrow-derived stem cells triggers a variety of neuronal biomolecules reported in our previous studies [23, 24, 27] suggesting that using MSC-CM against NDD can target important biological and functional processes to understand the mechanisms of their neuroprotection.

In this study, we examined the therapeutic efficacy of NI-hBMSC-CM treatment in the ROT-induced PD model on SH-SY5Y cells. Following treatment with 50% dilution of NI-hBMSC-CM for the last 24 h markedly diminished the toxic effects of ROT (0.5 *μ*M) for 48 h. Moreover, the majority of ROT-induced cells damaged and depleted lost their neurites, and their neurite network was found in SH-SY5Y cells; on the contrary, NI-hBMSC-CM treatment showed extending neurites that make contact with other neurites and cell bodies of neighboring cells. TH is the rate-limiting enzyme in the biosynthesis of the catecholamine neurotransmitters mainly responsible for tyrosine conversion into the dopamine precursor L-DOPA, which promotes the synaptic connections. The ROT-induced decline in TH activity in this study may be due to diminished substrate synthesis that correlates with the dopamine deficit that impairs dopamine synthesis, and its metabolism in neurons suggests the selective degeneration in PD [22]. Meanwhile, the increased TH expression after NI-hBMSC-CM treatment suggests the enhanced dopamine production results in the increased formation of synapses and neurotransmission.

*α*-syn at synaptic vesicles of neuronal cells have several important regulatory functions, including synaptic maintenance, mitochondrial homeostasis, proteasome function, dopamine metabolism, and chaperone activity [32]. The excess *α*-syn aggregation in the dopaminergic neurons induces a reduction in dopamine reuptake [33]. Folded naive *α*-syn is soluble and nontoxic; however, misfolded *α*-syn transform themselves into unstable dimers, which develop small to high molecular weight toxic insoluble oligomers in PD [34, 35]. These toxic oligomers of *α*-syn can be transmitted from diseased cells to healthy neurons and induce the conversion of native *α*-syn into toxic oligomeric species [36]. In addition, phosphorylation at serine129 (p-S129) of *α*-syn C terminus (95~140) induces a higher propensity for *α*-syn to aggregate [37] and diminished its affinity for vesicles [38]. Therefore, the Triton X-100-soluble and Triton X-100-insoluble cell protein fractions were analyzed for the expressions of oligomeric, dimeric, and monomeric p-S129 and total *α*-syn forms in Western blotting using paraformaldehyde and glutaraldehyde as membrane fixatives. In this study, the increased oligomers of insoluble p-S129 *α*-syn were in proportion to increased oligomers of insoluble total *α*-syn in ROT toxicity along with reduced soluble total *α*-syn oligomers suggesting that ROT increased the toxic *α*-syn aggregation with the progression of PD. Other studies also evidenced that the oligomeric *α*-syn positively correlated with the motor impairments in PD patients [39–41], and the increased p-S129 *α*-syn in PD patients is in the process of *α*-syn aggregation [42, 43].

In addition, the insoluble dimeric and monomeric p-S129 *α*-syn decreased in contrast with increased total *α*-syn dimeric and monomeric during ROT toxicity in this study suggest that insoluble p-S129 *α*-syn is mostly associated with oligomeric aggregation rather than dimeric or monomeric total *α*-syn. These increased Triton X-100-insoluble monomeric and dimeric total *α*-syn in this study indicate that inhibiting mitochondrial complex I by ROT affects the monomeric *α*-syn stability [44] that is involved in the initiation and the accumulation of total *α*-syn oligomers [45]. In addition, the unchanged ratio of soluble monomeric p-S129/total *α*-syn during ROT-induced toxicity suggests that the soluble p-S129 expression positively correlated with those of total soluble *α*-syn levels. Treatment of NI-hBMSC-CM significantly reduced the insoluble p-S129 oligomers and total *α*-syn level accumulation while increasing the soluble p-S129 and total *α*-syn during ROT toxicity. These results suggest that the insoluble p-S129 and total *α*-syn oligomers turn into soluble oligomers by enhancing the clearance of insoluble *α*-syn aggregation by NI-hBMSC-CM treatment and preserving the physiological functions of soluble *α*-syn monomers for the treatment of LBs in PD.

ROT-induced mitochondrial complex I dysfunction via disturbing mitochondrial dynamics subsequently impairs the cytoskeletal proteins [46], synaptic vesicle proteins [47], and axonal transport integrity [48]. The neurite outgrowth is mainly composed of cytoskeletal neurofilaments (NFs) and microtubules [49]. NFs have a role in forming and maintaining the axonal architecture and cargo transport within the neurons [50]. *α*-syn oligomers associated with decreased NF networks disrupts microtubule structures and axonal transport in neuronal functions [51]. The expression of NF-H (or NF200) appearing during axonal maturation is a major component of axonal outgrowth [52]. *β*3-Tubulin (or Tuj1 or TUBB3) is a microtubule-related neuronal cell marker [53]. In this study, the decreased expressions of NF-H and *β*3-tubulin during ROT toxicity were confirmed with previous findings that *α*-syn aggregation binds to NF-H and *β*3-tubulin [52, 54] that disturbs NF network integrity [55], and motor neurons were withdrawn from neuronal junction [56]. In addition, NF-H can directly bind the C terminal domain of tubulin and modulate its activity [57]. *β*3-Tubulin polymerization with *α*-syn to form an insoluble protein complex accumulates in the nerve terminals leading to neuronal dysfunction [58] with decreased *β*3-tubulin in NDD [59]. Treatment with NI-hBMSC-CM restored the NF-H and *β*3-tubulin expressions with increased soluble *α*-syn. These results suggest that NFs and microtubule networks are needed for progressive neurite formation, axonal outgrowth, and their cargo transport. This stabilized neuronal cytoskeleton integrity plays an important role in the *α*-syn regulation.

NeuN (or Fox-3 or RBFOX3) is a soluble nuclear protein associated with terminal neuronal differentiation used to evaluate neuronal cell loss in NDD [60]. Synaptophysin (SYP) is a membrane glycoprotein localized in presynaptic vesicles that have been used to access synaptic density [61] and neurotransmission [62]. However, *α*-syn aggregation in the presynaptic terminals defect synapse maintenance [56] and transmission [63]. In our study, the significant loss of NeuN during ROT toxicity along with decreased SYP protein expression indicates the synaptic neuronal degeneration because of *α*-syn accumulation at the synapse evidenced by others [64, 65], while NI-hBMSC-CM treatment increased the expressions of NeuN and SYP suggesting that the SH-SY5Y cells undergo neuronal differentiation with increased synaptic functions.

ROT inhibits mitochondrial ETC complex I causing accumulated electrons to generate intracellular reactive oxygen species (ROS), which triggers apoptosis activating neuronal death [66] in dopaminergic neurons [67, 68]. *α*-syn oligomerization and their accumulation exert a cytotoxic effect on neurons mainly linked to the apoptotic Bcl-2 family-related caspase pathways [69]. Bcl-2 family proteins consist of several proapoptotic and antiapoptotic members regulating the mitochondrial membrane integrity. The proapoptotic protein Bax resides in the cytosol. Bcl-2, one of the antiapoptotic proteins, reside in the outer mitochondrial membrane which inhibits cytochrome c release from mitochondria, stabilization of membrane potential, preservation of ATP production, and prevention of oxidative stress [70]. In this study, ROT-induced a significant increase in Bax protein that degraded Bcl-2 and Mcl-1 protein expressions. The Bax/Bcl-2 protein ratio is a better predictor of apoptotic cell death than the absolute concentrations of either Bax or Bcl-2 alone [71] which increased during ROT toxicity in SH-SY5Y cells, indicating the increased mitochondrial apoptosis. NI-hBMSC-CM treatment reduced the Bax/Bcl-2 ratio along with improved Mcl-1 in SH-SY5Y cells indicating the normalized mitochondrial functional protein expressions.

The translocation of Bax from the cytosol to the mitochondria promotes the release of cytochrome c from mitochondria to cytosol which was found to be induced by ROT that activates caspases [66]. Caspases are a family of proteases initially synthesized as inactive procaspases, which must undergo dimerization or oligomerization and then cleavage to become activated and promote apoptotic cell death [72]. In this study, ROT toxicity significantly decreased the procaspases of Cas-9, Cas-3, and Cas-7 evidences increasing active/cleaved caspase levels. Cytochrome c released from the mitochondria binds Apaf-1 and Cas-9 to form the “apoptosome” complex subsequently activating Cas-9 that can cleave and activate Cas-3 and Cas-7 [73]. Moreover, PARP-1 is cleaved into fragments that are detected specific to 89 kDa fragment appearing during apoptosis by the activation of Cas-3 and Cas-7 evidenced in this study. Excessive activation of caspases and PARP-1 triggers the apoptotic processes such as chromatin condensation, nuclear fragmentation, and cytoskeletal degradation [74, 75] depleting nicotinamide adenine dinucleotide (NAD) and ATP leading to cellular energy failure and cell death. Treatment of NI-hBMSC-CM against ROT toxicity controlled the prolevels of Cas-9, Cas-3, and Cas-7 and PARP-1 suggesting the inhibition on activation of intracellular apoptosis-related proteins. In addition, PARP-1 inhibitors can reduce cell death induced by *α*-syn [76], supporting that NI-hBMSC-CM treatment in this study diminished the ROT-induced mitochondrial defects, DNA damage, and apoptotic cell death.

## 5. Conclusion

**Figure 7:**
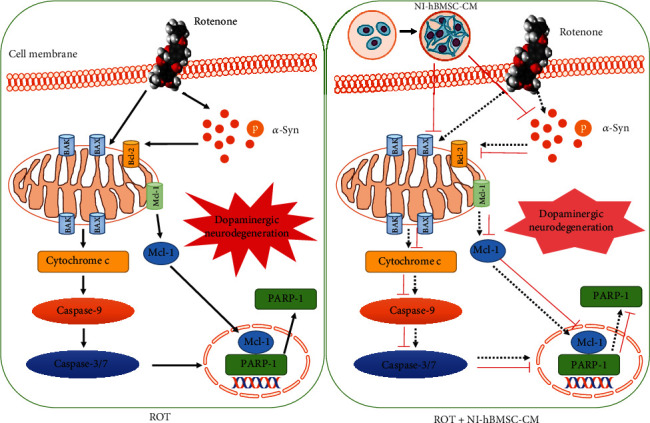
Diagrammatic representation of the proposed cell signaling activities of NI-hBMSC-CM against the ROT-induced PD-like model. Arrows denote ROT (black) and NI-hBMSC-CM (red).

In conclusion, cell survival and tyrosine hydroxylase protein expression were increased after NI-hBMSC-CM treatment by reducing *α*-syn phosphorylation, blocking toxic *α*-syn oligomers, and stabilizing the soluble *α*-syn monomers. NI-hBMSC-CM treatment improved the NF-H, *β*3-tubulin, NeuN, and SYP protein expressions along with the regulated Bax/Bcl-2 ratio and upregulated expression of procaspase-9, procaspase-3, and procaspase-7 and PARP-1. The above results evidenced that NI-hBMSC-CM has therapeutic effects against PD through inhibition of cell death, stabilization of *α*-syn monomers, promotion of neurogenesis, and lowering apoptosis as diagrammatically represented in Figure 7. The biomolecules released to the conditioned medium by bFGF and forskolin supplemented neural differentiation of hBMSC may be responsible for their neuroregenerative potential. Further studies in ROT-induced pathophysiological *α*-syn on an animal model of PD is needed to understand the novel therapeutic approach of neural differentiated conditioned medium.

## Data Availability

The datasets used and/or analyzed during this study are available from the corresponding authors upon reasonable request.

## Supplementary Materials

Supplementary Materials Supplementary Figure 1: SH-SY5Y cells were seeded as 5 × 10^4^ cells/mL of DMEM containing 1% FBS and used for experiments after overnight incubation. Cells incubated with the absence or presence of ROT (0.5 *μ*M) for 48 h were treated with hBMSC-CM or NI-hBMSC-CM at 100 or 50 or 25% during the last 24 h and assessed for morphological changes. Each picture is representative of three independent experiments. Supplementary Figure 2: (a) the experimental study plan. (b) SH-SY5Y cells were seeded as 5 × 10^4^ cells/mL of DMEM containing 1% FBS and used for experiments after overnight incubation. Cells incubated with the absence or presence of ROT (0.5 *μ*M) for 48 h were treated with hBMSC-CM (50%) or NI-hBMSC-CM (50%) during the last 24 h and assessed for morphological changes. Each picture is representative of three independent experiments. Supplementary Figure 3: SH-SY5Y cells were seeded as 5 × 10^4^ cells/mL of DMEM containing 1% FBS and used for experiments after overnight incubation. Cells with the absence or presence of ROT (0.5 *μ*M) for 48 h were treated with hBMSC-CM (50%) or NI-hBMSC-CM (50%) during the last 24 h and analyzed by Western blotting. Bar graphs represent fold changes in monomeric p-S129/total *α*-syn ratios from SDS-PAGE gels of 12% (a) or 8% (b) in Triton X-100-soluble fraction. The oligomeric, dimeric, and monomeric S129/total *α*-syn ratios from SDS-PAGE gels of 12% (c) or 8% (d) in Triton X-100-insoluble fraction. Data are the mean ± SEM of three independent experiments and analyzed by one-way analysis of variance (ANOVA) followed by Tukey's *post hoc* test. Statistical significance: ^a^compared with control; ^b^compared with ROT; ^∗^*p* < 0.05 and ^∗∗∗^*p* < 0.001. Supplementary Figure 4: SH-SY5Y cells were seeded as 5 × 10^4^ cells/mL of DMEM containing 1% FBS and used for experiments after overnight incubation. Cells with the absence or presence of ROT (0.5 *μ*M) for 48 h were treated with hBMSC-CM (50%) or NI-hBMSC-CM (50%) during the last 24 h and analyzed by Western blotting. The bar graphs represent the Bax/Bcl-1 ratio (a), pro-PARP-1/GAPDH (b), and cleaved PARP-1/GAPDH ratio (c). Each picture is representative of three independent experiments. Data are the mean ± SEM of three independent experiments and analyzed by one-way analysis of variance (ANOVA) followed by Tukey's *post hoc* test. Statistical significance: ^a^compared with control; ^b^compared with ROT; ^∗^*p* < 0.05 and ^∗∗∗^*p* < 0.001. Supplementary Table 1: Western blotting conditions and antibodies used in this study. Supplementary Figure 5: unedited images and their molecular weight markers for respective Western blots used in Figure 1(b) of this manuscript. Supplementary Figure 6: unedited images and their molecular weight markers for respective Western blots used in Figure 2(a) of this manuscript. Supplementary Figure 7: unedited images and their molecular weight markers for respective Western blots used in Figure 3(a) of this manuscript. Supplementary Figure 8: unedited images and their molecular weight markers for respective Western blots used in Figure 4 of this manuscript. Supplementary Figure 9: unedited images and their molecular weight markers for respective Western blots used in Figure 5 of this manuscript. Supplementary Figure 10: unedited images and their molecular weight markers for respective Western blots used in Figure 6 of this manuscript.
