# Supplementary material for: Therapeutic Effects of Conditioned Medium of Neural Differentiated Human Bone Marrow-Derived Stem Cells on Rotenone-Induced Alpha-Synuclein Aggregation and Apoptosis
Source: Stem Cells Int. 2021 Jan 22;2021:6658271. doi: 10.1155/2021/6658271 (PMC7847328; doi:10.1155/2021/6658271)
Supplement: Supplementary Materials — Supplementary Figure 1: SH-SY5Y cells were seeded as 5 × 10^4^ cells/mL of DMEM containing 1% FBS and used for experiments after overnight incubation. Cells incubated with the absence or presence of ROT (0.5 μM) for 48 h were treated with hBMSC-CM or NI-hBMSC-CM at 100 or 50 or 25% during the last 24 h and assessed for morphological changes. Each picture is representative of three independent experiments. Supplementary Figure 2: (a) the experimental study plan. (b) SH-SY5Y cells were seeded as 5 × 10^4^ cells/mL of DMEM containing 1% FBS and used for experiments after overnight incubation. Cells incubated with the absence or presence of ROT (0.5 μM) for 48 h were treated with hBMSC-CM (50%) or NI-hBMSC-CM (50%) during the last 24 h and assessed for morphological changes. Each picture is representative of three independent experiments. Supplementary Figure 3: SH-SY5Y cells were seeded as 5 × 10^4^ cells/mL of DMEM containing 1% FBS and used for experiments after overnight incubation. Cells with the absence or presence of ROT (0.5 μM) for 48 h were treated with hBMSC-CM (50%) or NI-hBMSC-CM (50%) during the last 24 h and analyzed by Western blotting. Bar graphs represent fold changes in monomeric p-S129/total α-syn ratios from SDS-PAGE gels of 12% (a) or 8% (b) in Triton X-100-soluble fraction. The oligomeric, dimeric, and monomeric S129/total α-syn ratios from SDS-PAGE gels of 12% (c) or 8% (d) in Triton X-100-insoluble fraction. Data are the mean ± SEM of three independent experiments and analyzed by one-way analysis of variance (ANOVA) followed by Tukey's post hoc test. Statistical significance: acompared with control; bcompared with ROT; ∗p < 0.05 and ∗∗∗p < 0.001. Supplementary Figure 4: SH-SY5Y cells were seeded as 5 × 10^4^ cells/mL of DMEM containing 1% FBS and used for experiments after overnight incubation. Cells with the absence or presence of ROT (0.5 μM) for 48 h were treated with hBMSC-CM (50%) or NI-hBMSC-CM (50%) during the last 24 h and analyzed by Western bl [file 6658271.f1.pdf]

Supplementary Materials for

“Therapeutic Effects of Conditioned Medium of Neural Differentiated Human Bone Marrow-Derived Stem Cells on Rotenone-Induced alpha-Synuclein Aggregation and Apoptosis”

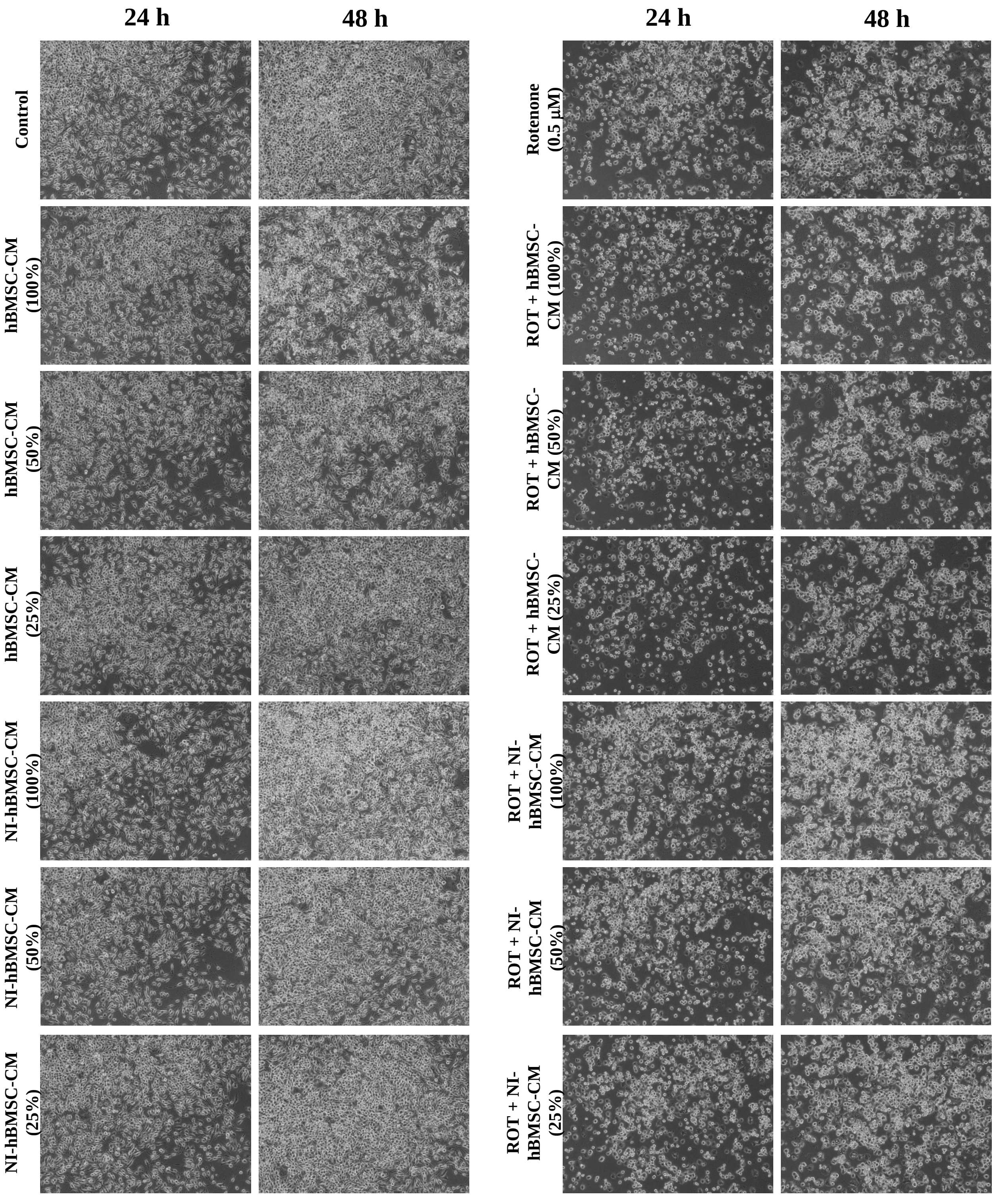

SUPPLEMENTARY FIGURE 1: SH-SY5Y cells were seeded as 5×10^4 cells/ml of DMEM containing 1% FBS and used for experiments after overnight incubation. Cells incubated with the absence or presence of ROT (0.5 μM) for 48 h were treated with hBMSC-CM or NI-hBMSC-CM at 100 or 50 or 25% during the last 24 h and assessed for morphological changes. Each picture is representative of three independent experiments.

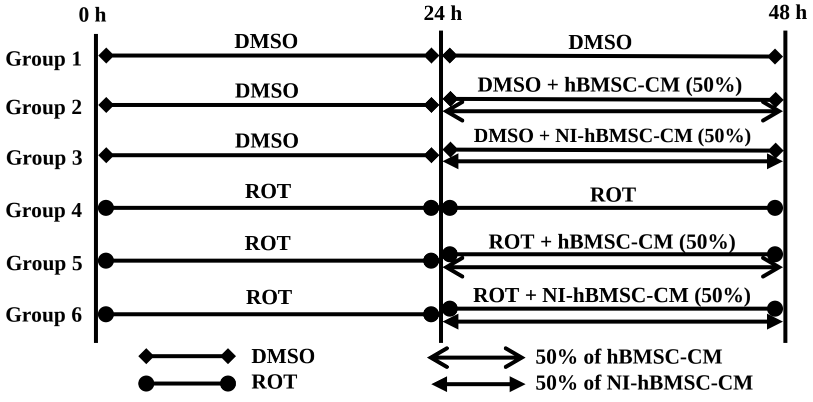

(a)

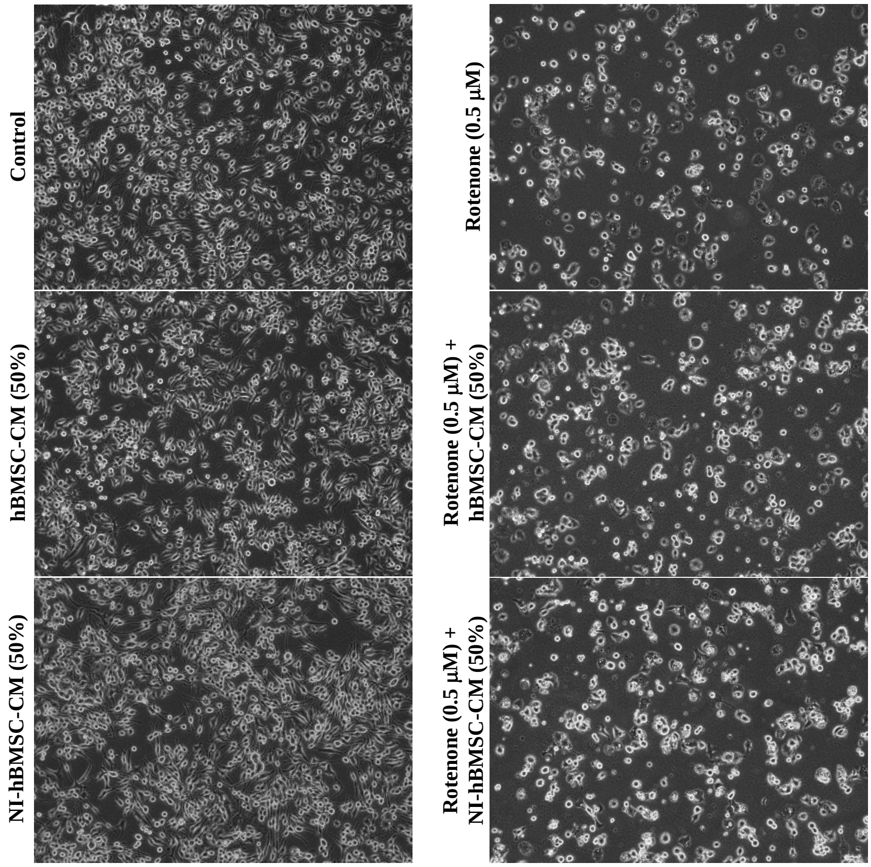

(b)

SUPPLEMENTARY FIGURE 2: (a) The experimental study plan. (b) SHSY5Y cells were seeded as 5×10^4 cells/ml of DMEM containing 1% FBS and used for experiments after overnight incubation. Cells incubated with the absence or presence of ROT (0.5μM) for 48 h were treated with hBMSC-CM (50%) or NI-hBMSC-CM (50%) during the last 24 h and assessed for morphological changes. Each picture is representative of three independent experiments.

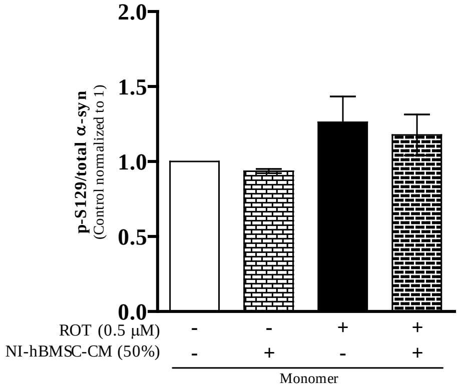

(a)

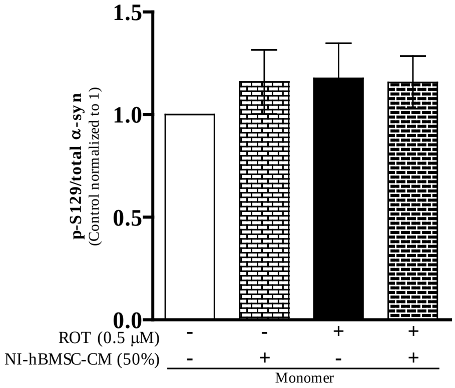

(b)

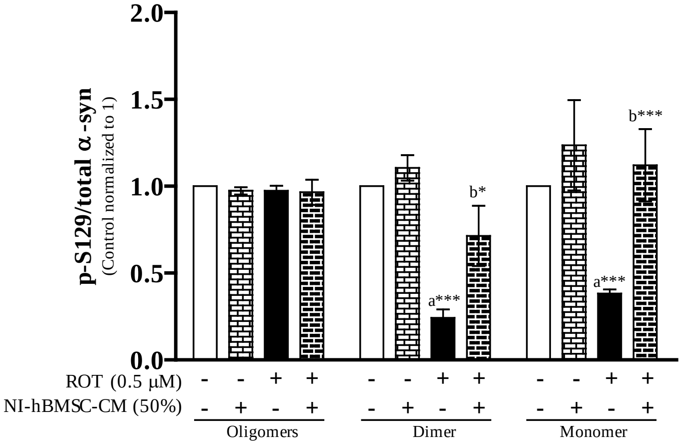

(c)

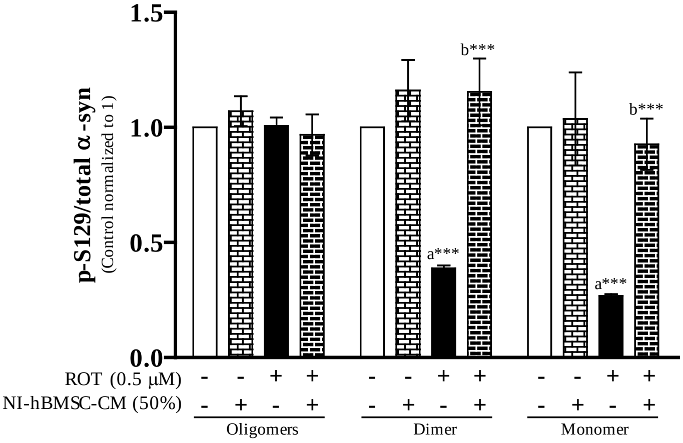

(d)

SUPPLEMENTARY FIGURE 3: SH-SY5Y cells were seeded as 5×10^4 cells/ml of DMEM containing 1% FBS and used for experiments after overnight incubation. Cells with absence or presence of ROT (0.5 μM) for 48 h were treated with hBMSC-CM (50%) or NI-hBMSC-CM (50%) during the last 24 h and analyzed by Western blotting. Bar graphs represents fold changes in monomeric p-S129/total α-syn ratios from SDS-PAGE gels of 12% (a) or 8% (b) in Triton X-100-soluble fraction. The oligomeric, dimeric and monomeric S129/total α-syn ratios from SDS-PAGE gels of 12% (c) or 8% (d) in Triton X-100-insoluble fraction. Data are mean ± SEM of three independent experiments and analyzed by one-way of variance (ANOVA) followed by Tukey's *post hoc* test. Statistical significance: [a]-compared with control; [b]-compared with ROT; **p*<0.05 and ****p*<0.001.

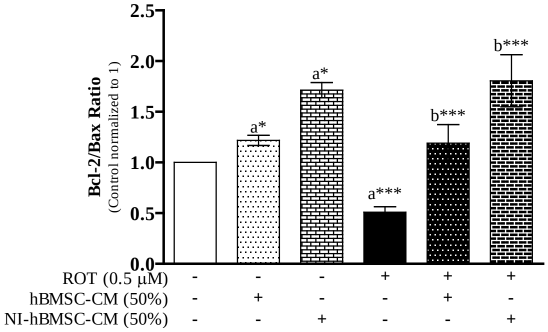

(a)

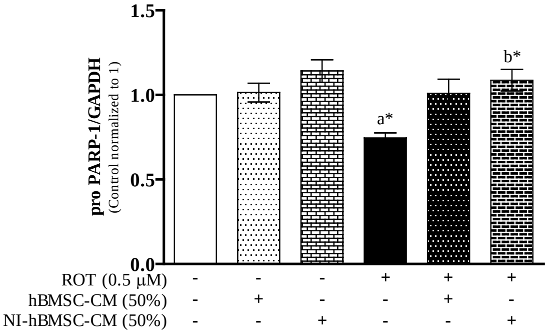

(b)

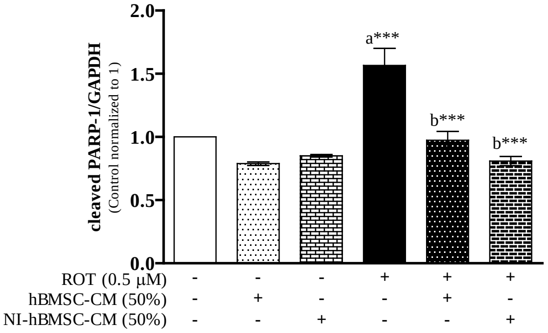

(c)

SUPPLEMENTARY FIGURE 4: SH-SY5Y cells were seeded as 5×10^4 cells/ml of DMEM containing 1% FBS and used for experiments after overnight incubation. Cells with absence or presence of ROT (0.5 μM) for 48 h were treated with hBMSC-CM (50%) or NI-hBMSC-CM (50%) during the last 24 h and analyzed by Western blotting. The bar graphs represents for Bax/Bcl-1 ratio (a), pro-PARP-1/GAPDH (b) and cleaved PARP-1/GAPDH ratio (c). Data are mean ± SEM of three independent experiments and analyzed by one-way of variance (ANOVA) followed by Tukey's *post hoc* test. Statistical significance: [a]-compared with control; [b]-compared with ROT; *p<0.05 and ***p<0.001.

SUPPLEMENTARY TABLE 1: Western blotting conditions and antibodies used in this study.

Antibody Name	Host, MW Details	Company	Cat. No.	Dilution
**Primary Antibodies:**				
Tyrosine hydroxylase	Rabbit pAb, 62 kDa	Millipore	AB152	1:1,000
p-S129 α-synuclein	Rabbit mAb, 18 kDa	Abcam	ab51253	1:1,000
total α-synuclein	Rabbit mAb, 18 kDa	Abcam	ab212184	1:1,000
Neurofilament-H	Mouse mAb, 180~200 kDa	Cell Signaling	#2836	1:1,000
β3-tubulin	Rabbit mAb, 55 kDa	Cell Signaling	#5568	1:1,000
Neuronal Nuclei	Mouse mAb, 46~48 kDa	Millipore	MAB377	1:1,000
Synaptophysin	Mouse mAb, 38~48 kDa	Santa Cruz	sc-17750	1:2,000
Bax	Rabbit pAb, 23 kDa	Santa Cruz	sc-493	1:500
Bcl-2	Rabbit pAb, 26 kDa	Santa Cruz	sc-492	1:500
Mcl-1	Rabbit mAb, 40 kDa	Cell Signaling	#94296	1:1,000
Caspase-9	Mouse mAb, pro=47, cleaved=37,35 kDa	Cell Signaling	#9508	1:1,000
Caspase-3	Rabbit mAb, pro=35, cleaved=17,19 kDa	Cell Signaling	#9665	1:1,000
Caspase-7	Rabbit mAb, Pro=35, cleaved=20 kDa	Cell Signaling	#12827	1:1,000
PARP	Rabbit pAb, Pro=116, cleaved=89 kDa	Cell Signaling	#9542	1:1,000
GAPDH	Rabbit pAb, 37 kDa	Santa Cruz	sc-25778	1:2,000
β-actin	Mouse mAb, 43 kDa	Santa Cruz	sc-47778	1:2,000
**Secondary Antibodies:**				
Anti-rabbit IgG, HRP-linked antibody		Cell Signaling	#7074	1:1,000 ~1:2,000
Anti-mouse IgG, HRP-linked antibody		Cell Signaling	#7076	1:1,000 ~1:2,000

Western blotting conditions:

SDS-PAGE Gel Percentages

8% = TH, p-S129 α-syn, total α-syn, NF-H, PARP

12% = p-S129 α-syn, total α-syn, β3-tubulin, NeuN, SYP

13 or 14% = Bax, Bcl-2, Mcl-1, Cas-9, -3, -7.

SDS-PAGE Gel Running:

80~100 V for 100~120 min

SDS-PAGE Gel Transfer Times to Nitrocellulose Membrane:

8% = 250 mA for 90 min

12% = 200 mA for 65 min

13 or 14% = 200 mA for 60 min

For Figure 1(b)

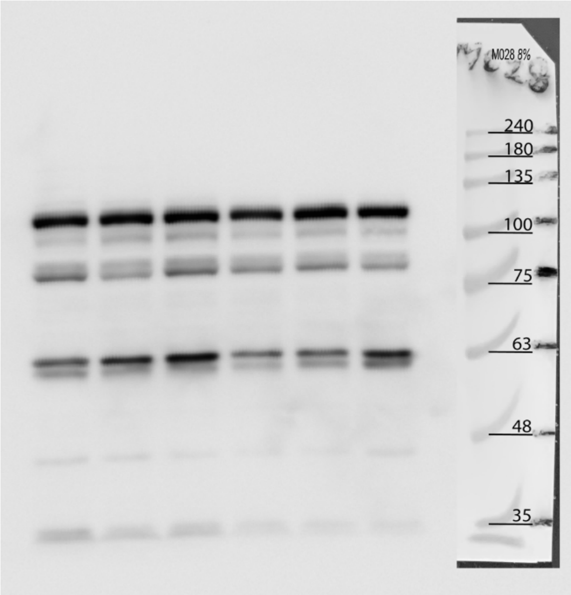

TH (62 kDa) →

**ROT** (0.5 μM)	-	-	-	+	+	+
**hBMSC-CM** (50%)	-	+	-	-	+	-
**NI-hBMSC-CM** (50%)	-	-	+	-	-	+

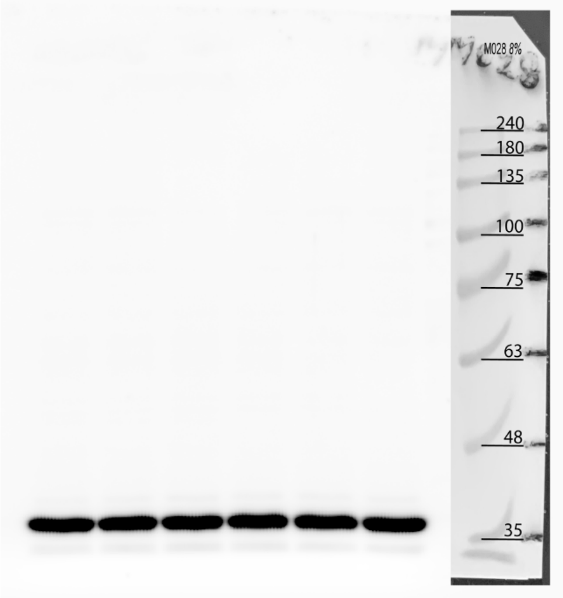

GAPDH (37 kDa) →

**ROT** (0.5 μM)	-	-	-	+	+	+
**hBMSC-CM** (50%)	-	+	-	-	+	-
**NI-hBMSC-CM** (50%)	-	-	+	-	-	+

SUPPLEMENTARY FIGURE 5: Unedited images and their molecular weight markers for respective Western blots used in **FIGURE 1(b)** of this manuscript.

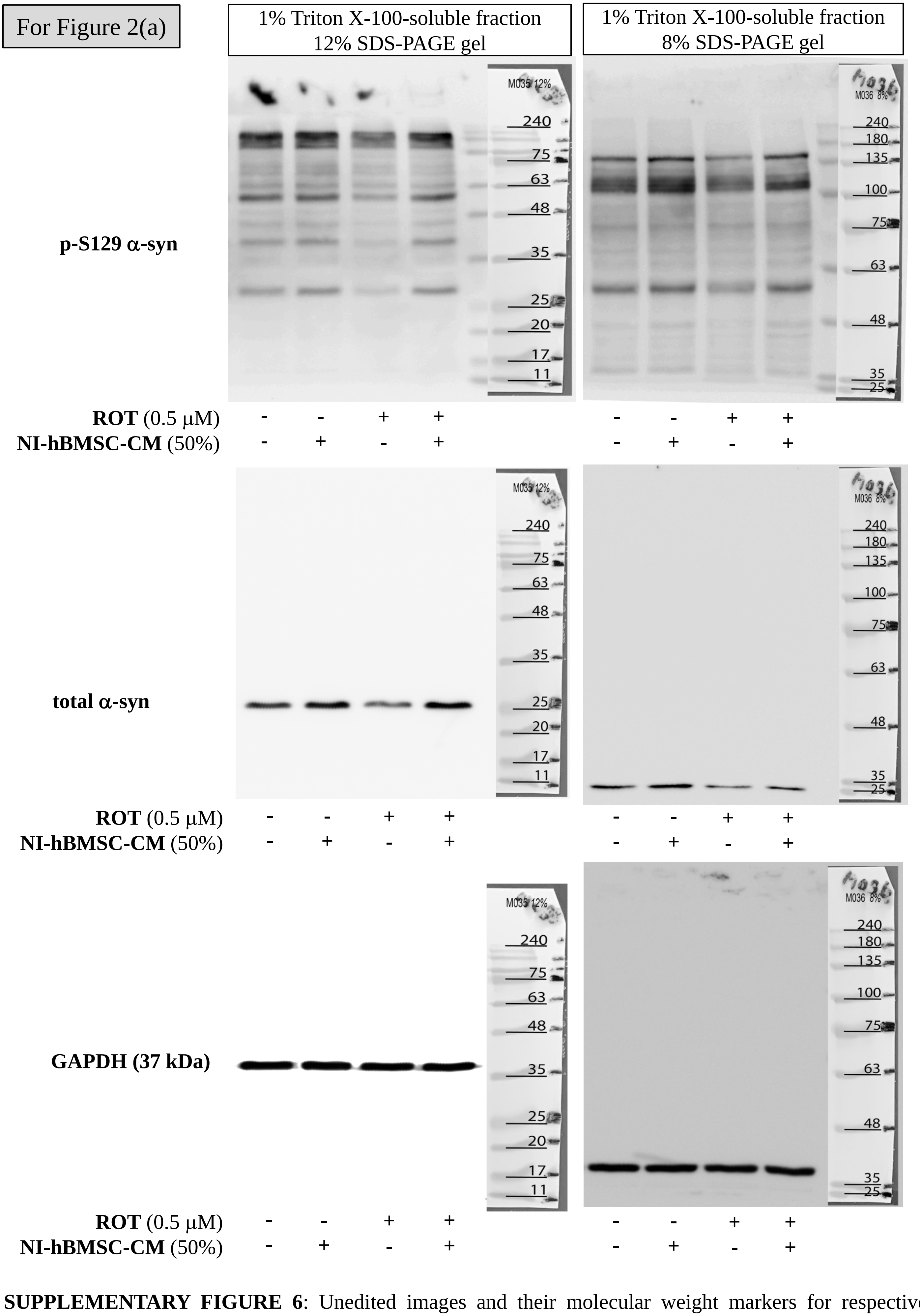

SUPPLEMENTARY FIGURE 6: Unedited images and their molecular weight markers for respective Western blots used in **FIGURE 2(a)** of this manuscript.

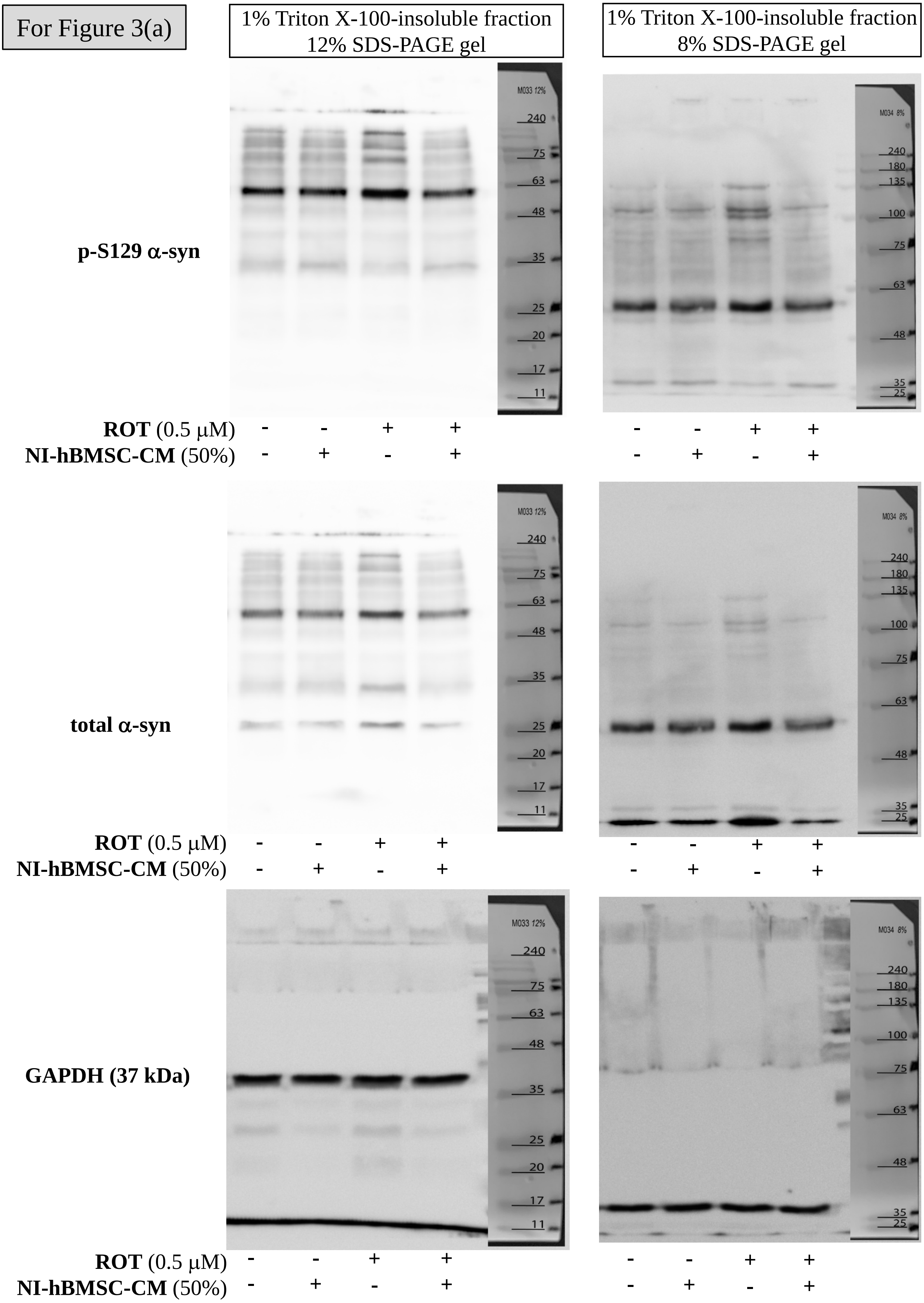

SUPPLEMENTARY FIGURE 7: Unedited images and their molecular weight markers for respective Western blots used in **FIGURE 3(a)** of this manuscript.

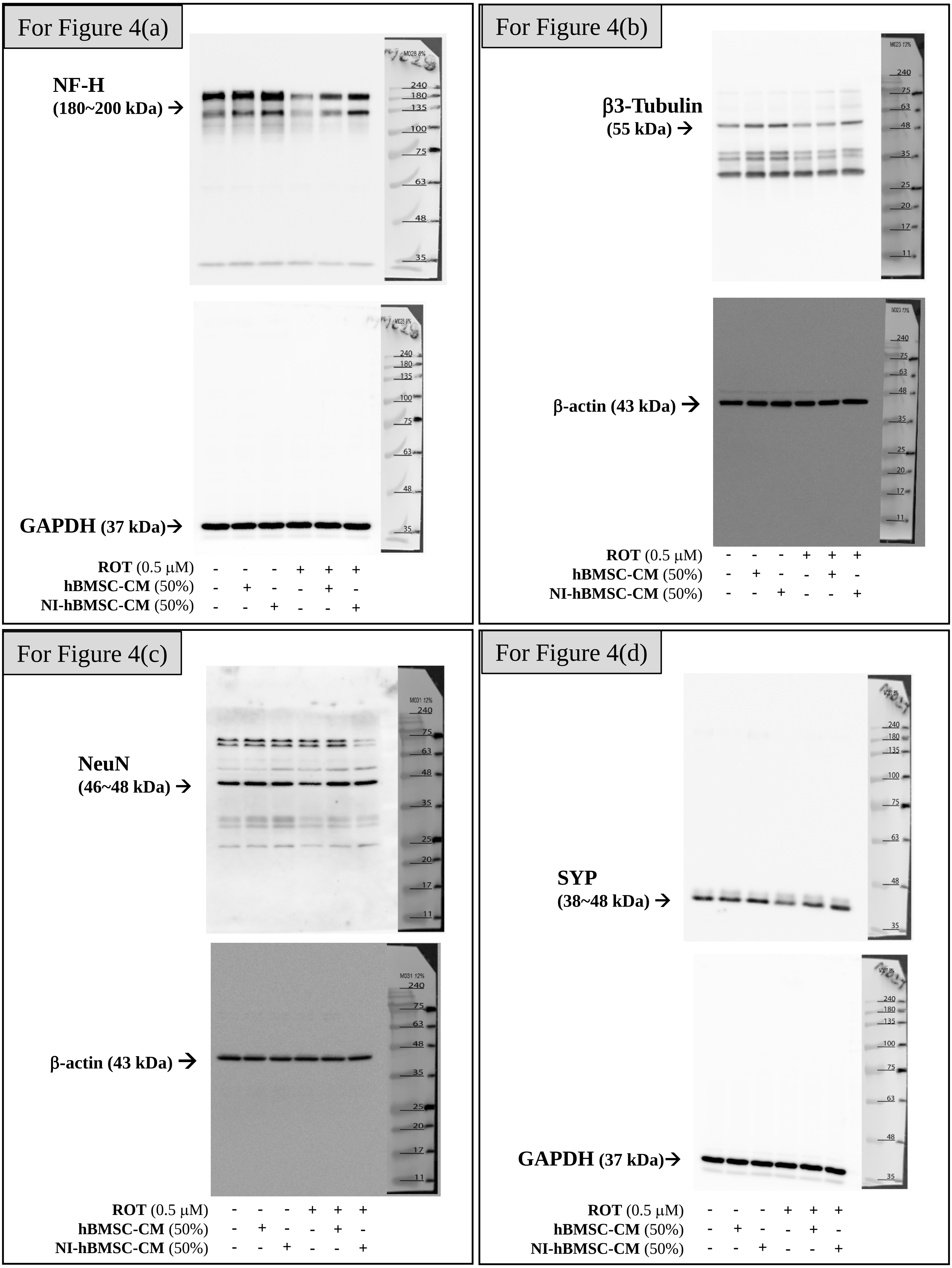

SUPPLEMENTARY FIGURE 8: Unedited images and their molecular weight markers for respective Western blots used in **FIGURE 4** of this manuscript.

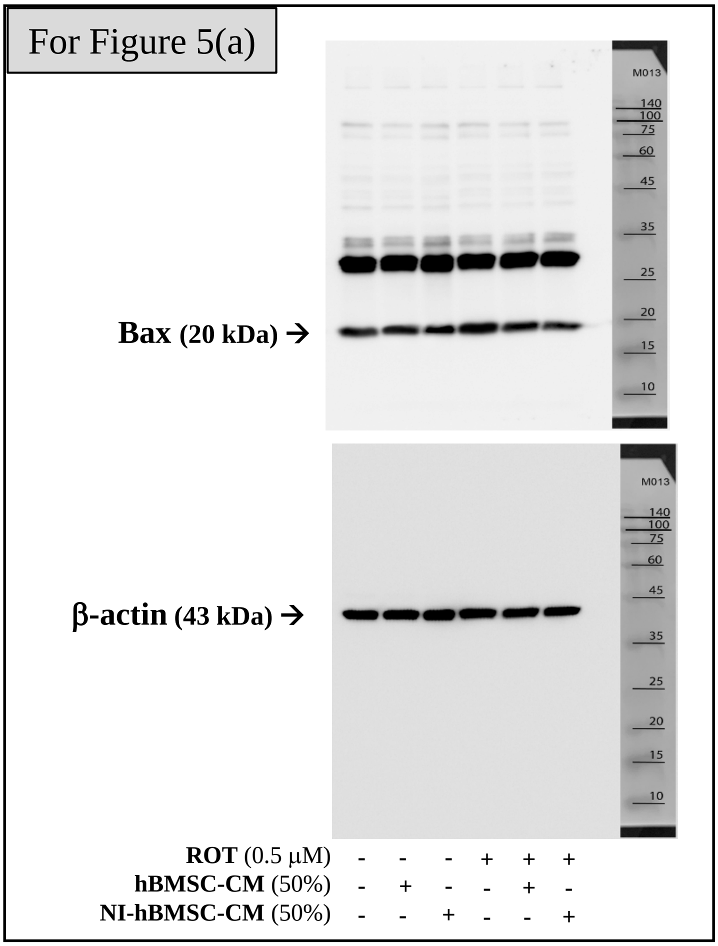

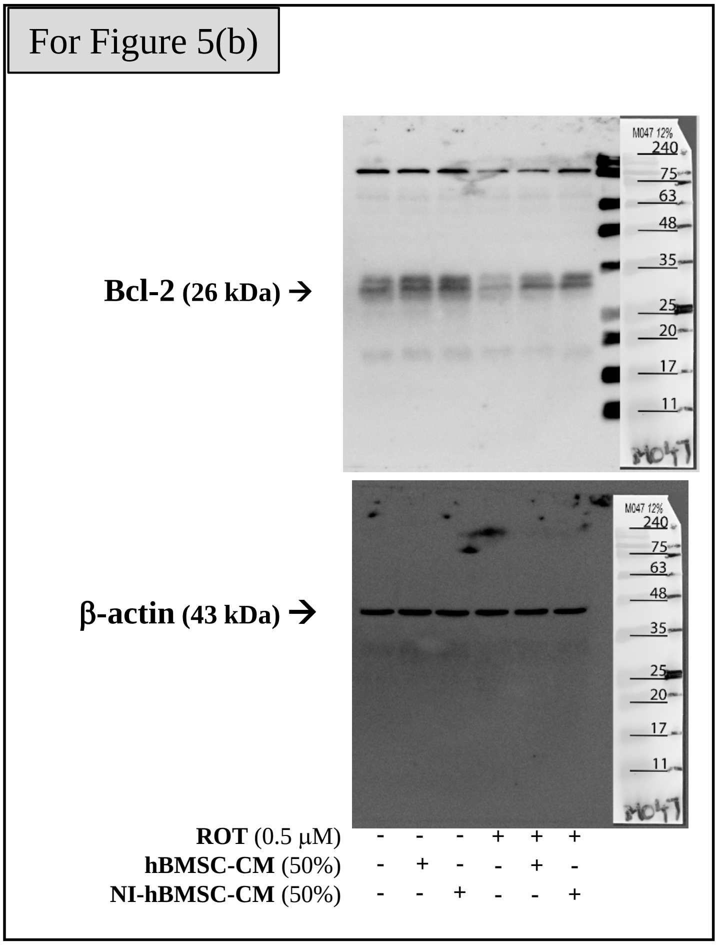

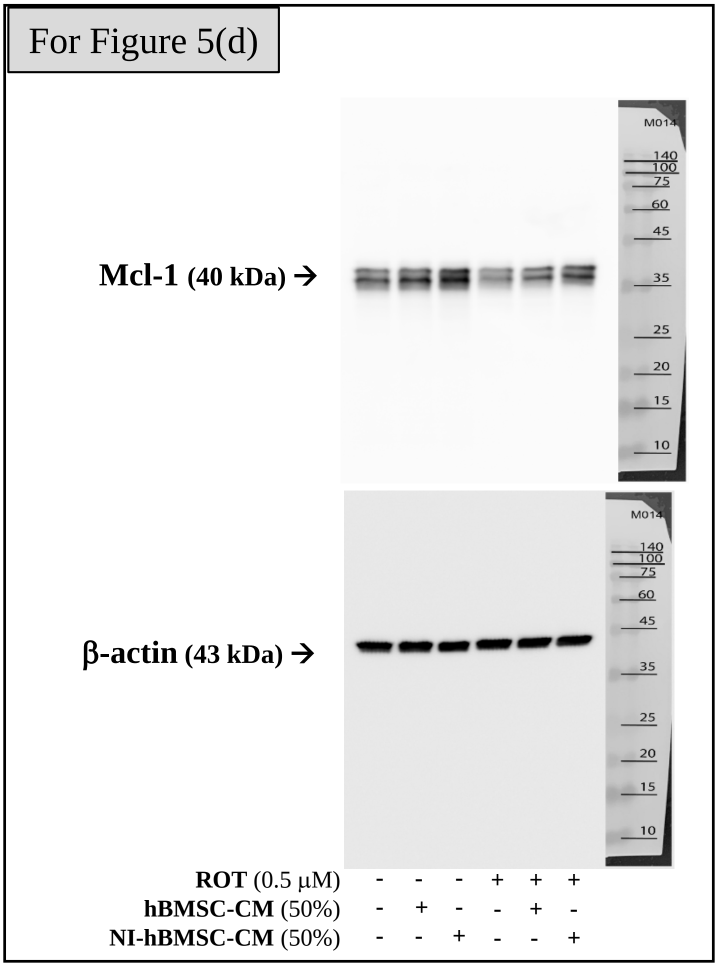

SUPPLEMENTARY FIGURE 9: Unedited images and their molecular weight markers for respective Western blots used in **FIGURE 5** of this manuscript.

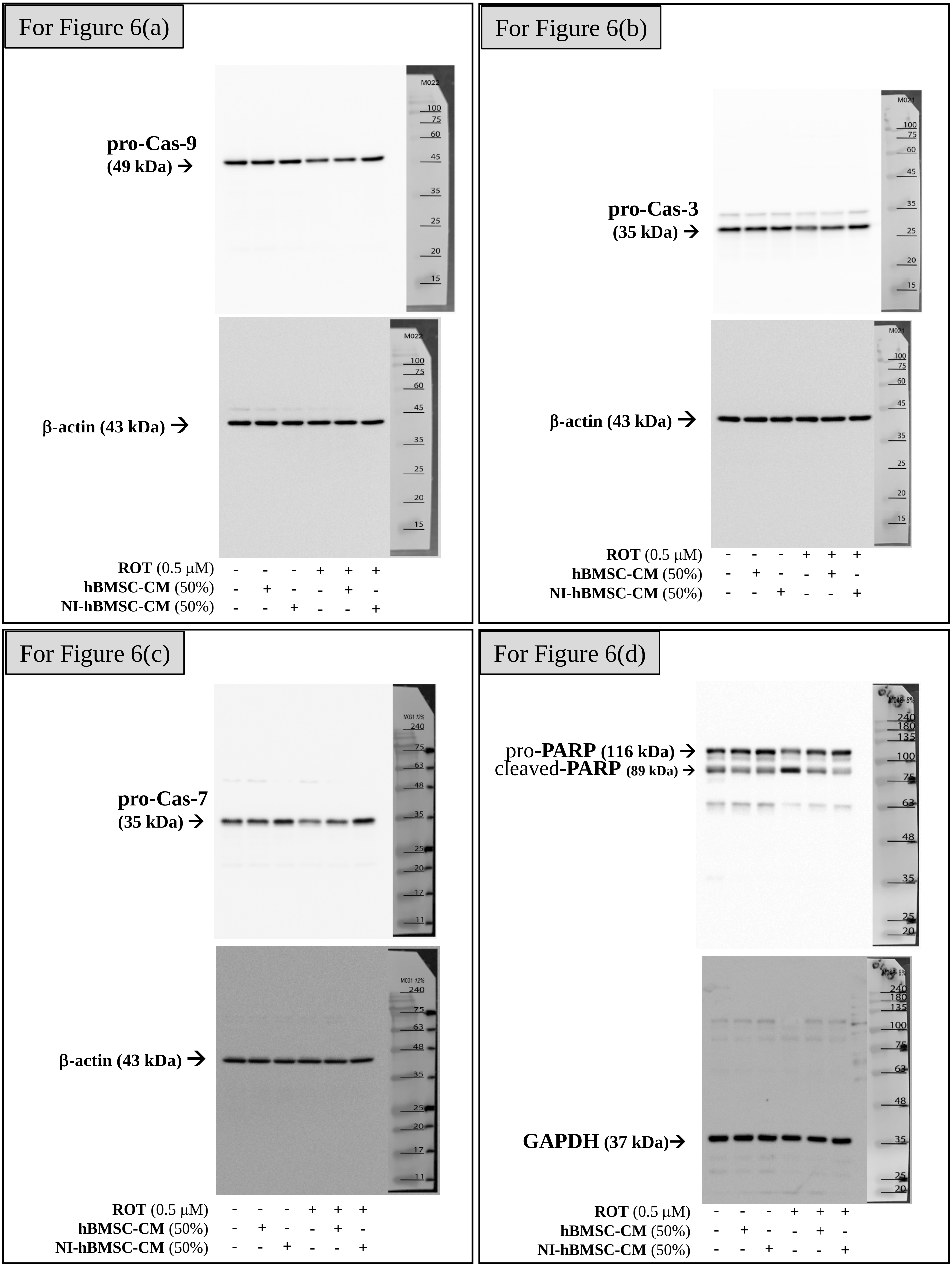

SUPPLEMENTARY FIGURE 10: Unedited images and their molecular weight markers for respective Western blots used in **FIGURE 6** of this manuscript.
